# Study on the Effect of the Pre-Forming of 22MnB5 Steel in Indirect Hot Stamping

**DOI:** 10.3390/ma16103739

**Published:** 2023-05-15

**Authors:** Ziming Tang, Zhengwei Gu, Yi Li, Xin Li, Ge Yu, Lingling Yi

**Affiliations:** School of Materials Science and Engineering, Key Laboratory of Automobile Materials, Jilin University, Changchun 130022, China

**Keywords:** indirect hot-stamping technology, ultra-high-strength steel, pre-forming, microstructure

## Abstract

Based on the indirect hot-stamping test system, the effect of pre-forming on the microstructure evolution (grain size, dislocation density, martensite phase transformation) and mechanical properties of the blank in indirect hot stamping is systematically studied using ultra-high-strength steel 22MnB5. It is found that the average austenite grain size slightly decreases with the increase in pre-forming. After quenching, the martensite also becomes finer and more uniformly distributed. Although the dislocation density after quenching slightly decreases with the increase in pre-forming, the overall mechanical properties of the quenched blank are not greatly affected by pre-forming under the combined effect of the grain size and dislocation density. Then, this paper discusses the effect of the pre-forming volume on part formability in indirect hot stamping by manufacturing a typical beam part. According to the numerical simulations and experimental results, when the pre-forming volume increases from 30% to 90%, the maximum thickness thinning rate of the beam part decreases from 30.1% to 19.1%, and the final beam part has better formability and more uniform thickness distribution results when the pre-forming volume is 90%.

## 1. Introduction

**Figure 1:**
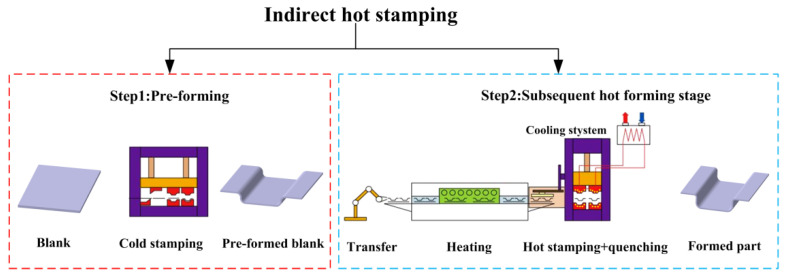
Indirect hot-stamping process.

In the context of promoting energy saving and environmental protection in the automotive field, ultra-high-strength steel hot-stamping technology has received much attention from the industry as an effective method to achieve the high-strength and lightweight goals of parts [1,2,3]. Hot-stamping technology can be divided into direct and indirect hot-stamping technology [4]. Direct hot-stamping technology means that the blank is directly sent to the heating furnace for austenitization and then transferred to the dies with cooling water channels for stamping and quenching to obtain formed parts with ultra-high strength [5,6]. The blank is transformed from the original pearlite and ferrite to austenite at high temperature and then achieves ultra-high strength by martensite phase transformation during quenching. Direct hot-stamping technology is often used to manufacture automobile A-pillars and B-pillars, as well as other ultra-high-strength reinforcing components [7,8]. As shown in Figure 1, indirect hot-stamping technology can be divided into two stages [9]. First is the pre-forming stage, which requires the blank to be partially cold stamped at room temperature, where the blank will develop some initial shape and forming depth. This paper defines the second stage as subsequent hot forming, which means the pre-formed blank is transferred to the heating furnace to achieve austenitization and then transferred to the dies with cooling water channels for further stamping and quenching. In contrast to direct hot-stamping technology, indirect hot-stamping technology can form parts with more complex cross-section shapes, larger forming depths, and larger thicknesses, such as the middle channel parts in automobiles [10,11].

At present, the automobile industry has generally mastered direct hot-stamping technology, and there are extensive studies about direct hot stamping. Skowronek et al. presented a comprehensive study of direct hot stamping by manufacturing an automotive door beam part as an example. Based on the process design and numerical simulation, the door beam part was well manufactured with the expected performance [12]. Wróbel et al. designed an automatic direct hot-stamping line for a body component in an automobile with the Industry 4.0 concept [13]. Chen et al. took 1.2 mm thickness B1500HS steel as the research object and optimized the process parameters in direct hot stamping in a series of experiments [14]. Xu et al. studied the softening behavior of 22MnB5 steel during direct hot stamping in a series of isothermal hot compression tests and modified the constitutive model of the material [15].

Only very few studies have focused on indirect hot-stamping technology. Seo et al. compared the determined formability of the direct and indirect hot-stamping technology by cupping tests and confirmed that the forming depth of the blank after indirect hot stamping is deeper than direct hot stamping [16]. Park et al. used indirect hot-stamping technology to form a coupled torsion beam axle and confirmed that the part has acceptable forming quality after indirect hot stamping [17]. Min et al. studied indirect hot-stamping technology by pre-strain tests, but their paper only discussed the martensite microstructure of the blank after quenching and lacked analyses of other microstructures and data [18]. In general, these references indicate that indirect hot stamping can improve part forming accuracy and formability more than direct hot stamping, but there is a lack of other systematic conclusions about this technology. Indirect hot stamping is more complicated since it has both cold-stamping and hot-stamping processes. The interior grain morphology and mechanical property of the blank will change when the blank is deformed during the pre-forming stage. Thus, the pre-forming stage is important because the condition of the blank after pre-forming will directly affect the results of subsequent hot forming. However, few researchers have investigated the evolutionary processes of how the pre-deformed blank changes during subsequent hot forming and whether pre-forming will affect the microstructure and mechanical properties of the final formed part. Furthermore, the concept of the “pre-forming volume” in the pre-forming stage has not been fully defined. Theoretically, the more the blank is formed during the pre-forming stage, the less it is formed during subsequent hot forming, and the more evenly the temperature and thickness are distributed during subsequent hot forming. However, due to the material properties of ultra-high-strength steel at room temperature, large pre-forming volumes will cause defects such as cracks, wrinkles, and more springback, these defects will continue to affect the final part’s formability. Moreover, when the part is more complex or has a larger forming depth, pre-forming cannot reach 100% of the final part size. Thus, to ensure the formability of the pre-formed blank and also satisfy the hot-stamping requirements, the pre-forming volume should be in a suitable range.

To sum up, current research on indirect hot-stamping technology is still not sufficient, and there is a lack of relevant basic theory and accurate data to better develop this technology. Thus, in this work, the effect of pre-forming on microstructure evolution, mechanical properties, and part formability in indirect hot stamping was systematically studied by both finite-element (FE) simulations and experimental methods.

## 2. Effect of Pre-Forming on the Material in Indirect Hot Stamping

### 2.1. Materials and Methods

The material used in this work is hot-rolled pickled steel 22MnB5 with a thickness of 2 mm provided by Baosteel Group. The chemical composition of the material is presented in Table 1.

**Figure 2:**
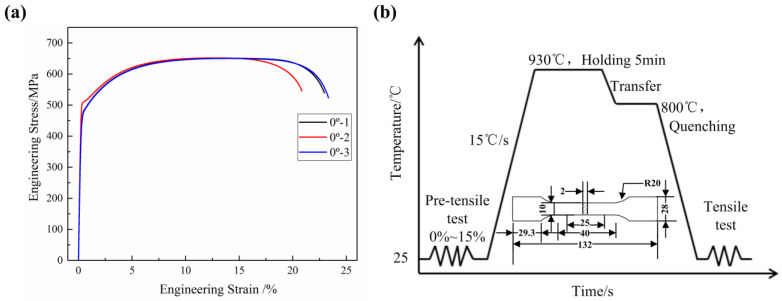
(**a**) Engineering stress–strain curve of the delivered blank at RD direction and (**b**) schematic diagram of the test.

First, the uniaxial tensile test of the blank in the rolling direction (RD) shows that the average elongation is 22.39% (Figure 2a). The specimens experience necking when the elongation is greater than 16%. Secondly, an experimental test system was designed to imitate indirect hot stamping using the RD blank, as shown in Figure 2b. The blanks were first cut into 60 mm wide rectangles and pre-tensiled for different elongations of 0%, 3%, 5%, 8%, 10%, and 15%. This step was similar to the pre-forming stage. The blank underwent different deformations and was represented by different elongations. Then, the tensile specimens were cut in the middle of the pre-tensiled blanks and subjected to the subsequent hot-forming stage. The heat treatment parameters included heating the specimens to 930 °C followed by holding for 5 min to achieve complete austenitization. Then, the specimens were quenched in cold water at 800 °C. Finally, the quenched specimens were subjected to a second uniaxial tensile test to obtain the quenching mechanical properties. Each elongation was tested four times. One specimen was used to cut small samples in the gauge length area with 10 mm × 10 mm for the microstructure characterization and hardness tests, and three specimens were used for the second tensile test to obtain the average quenching mechanical properties.

The microstructure and grain morphology in each stage were observed with electron backscattered diffraction (EBSD), scanning electron microscopy (SEM), transmission electron microscopy (TEM), and X-ray diffraction (XRD). The samples were etched with an aqueous picric acid solution containing a small amount of sodium tridecyl benzene sulfonate to restore the austenite grain boundaries and then observed with an Olympus microscope. The etching process was carried out in 80 °C hot water for about 15 min. The samples were etched with 4% nitric acid alcohol solution to observe the martensite structure, and the samples were electrolytically polished in 9% perchloric acid alcohol solution for EBSD observation. Both microstructures were observed using a JSM-7900F scanning electron microscope. The EBSD observation was performed at 500 magnification. The machine was operated at 20 kV. The scanning step of the samples at room temperature was 0.5 μm, the scanning step of the quenched samples was 0.3 μm, and the observation surface was RD × ND. The TEM samples were prepared by mechanical thinning to 30 μm~50 μm followed by final ion-beam thinning. A Φ3 mm film was cut with a punching machine, electrolytically polished in 5% perchloric acid alcohol solution, and a JEOL 2100 transmission electron microscope was used to observe the internal dislocation structure. The surfaces of the samples were ground and polished for the XRD test. The XRD patterns were recorded in the 2θ range of 30° to 90° in reflection mode using a Rigaku D/Max 2500PC X-ray diffractometer with Cu radiation. The machine was operated at 35 kV and 25 mA with a 0.02° step size.

### 2.2. Microstructure Evolution

**Figure 3:**
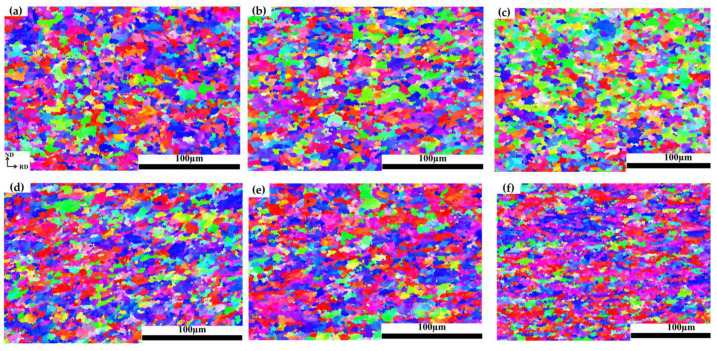
IPF maps of the specimens with different elongations at room temperature: (**a**) 0%; (**b**) 3%; (**c**) 5%; (**d**) 8%; (**e**) 10%; (**f**) 15%.

Figure 3 shows the inverse pole figure (IPF) maps of the specimens with different elongations after pre-tensile deformation at room temperature. When the elongation is 0%, the grain morphology is mainly equiaxed grain, and with the increase in elongations, the grains are elongated along the tensile direction. When the elongation is 15% (Figure 3f), the grains are elongated and flattened, and the grain boundaries become fuzzy and fibrous. With the increase in elongations, the average grain size decreases from 6.2 μm to 5.6 μm.

**Figure 4:**
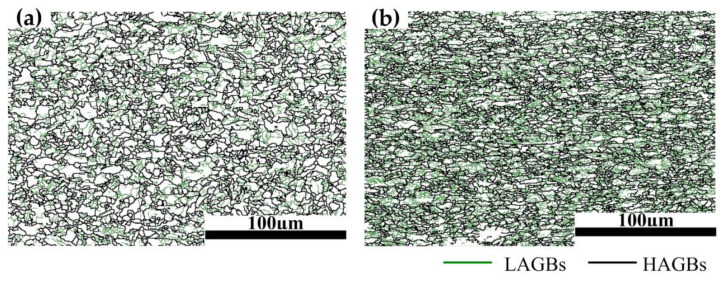
Grain boundary map of the specimens at room temperature: (**a**) 0%; (**b**) 15%.

Figure 4 shows the grain boundary map of the 0% specimen and 15% specimen at room temperature. The low-angle grain boundaries (LAGBs) are represented by green solid lines, and the high-angle grain boundaries (HAGBs) are represented by black solid lines. According to the statistical results, the percentage of LAGBs increases from 23.8% to 36.4% with the increase in elongations. The LAGBs are formed because of the increase in dislocation density inside the specimens after pre-tensile deformation, where the larger amount of deformation causes more LAGBs.

As a high-temperature phase in the heating process of 22MnB5, the austenite grain characteristics, including its homogenization degree, grain morphology, and grain size, will affect the mechanical properties of the final quenched blank. After the austenite crystal nucleus forms, the grain size mainly depends on the heat treatment process, such as the heating speed, heating temperature, and holding time. In addition, it depends on the original structure and chemical composition of the blank.

**Figure 5:**
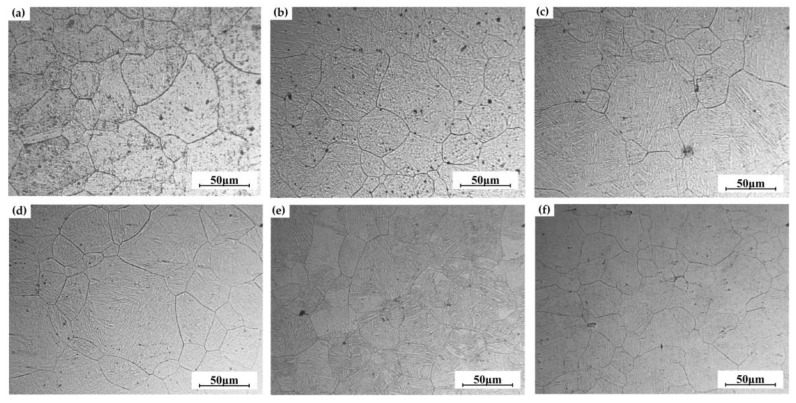
Austenite grain distribution results of the quenched specimens with different elongations: (**a**) 0%; (**b**) 3%; (**c**) 5%; (**d**) 8%; (**e**) 10%; (**f**) 15%.

Figure 5 shows the austenite grain distribution results of the quenched specimens with different elongations. As shown in Figure 5a, when the elongation is 0%, the austenite grain distribution is relatively uniform. When the elongation is 3% to 8% (Figure 5b–d), there are individual coarse austenite grains in the grain area, but the growth is not very large. When the elongation exceeds 10%, the austenite grain distribution returns to a more uniform state. When the elongation is in the range of 3% to 8%, it is similar to the critical degree of deformation in recrystallization. The blank undergoes uneven deformation, and thus individual coarse grains appear in the austenite grain area. When the elongation exceeds 10%, the austenite grain distribution becomes more uniform. The average austenite grain size slightly decreases from 44.5 μm to 40.5 μm with the increase in elongations. According to the early results, the average grain size at room temperature decreases with the increase in elongations; thus, during the same heating process, when the heating temperature is over A_c3_, the austenite nuclei are uniformly formed, and after the same holding time, the austenite grain distribution is also more uniform. It can be seen that with the increase in elongations, the austenite grains can be slightly refined and the austenite distribution is more uniform.

**Figure 6:**
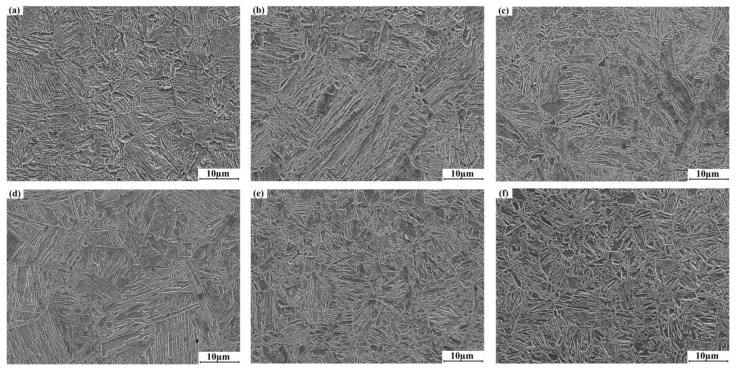
Martensite distribution results of the quenched specimens with different elongations: (**a**) 0%; (**b**) 3%; (**c**) 5%; (**d**) 8%; (**e**) 10%; (**f**) 15%.

Figure 6 shows the microstructure distribution results of the quenched specimens with different elongations. After quenching, the microstructures of the quenched specimens are full martensite. The martensite growth will not exceed the austenite grain boundaries; therefore, the austenite grain size directly determines the martensite size. When the elongation is 3% to 8% (Figure 6b–d), based on the austenite grain results, the long and short axes of martensite after the phase transformation of the individual coarse austenite grains seem relatively large. Overall, the martensite seems finer and more uniformly distributed with the increase in elongations, and the average length of the martensite is about 10 μm.

### 2.3. Dislocation Density

The dislocation density inside the blank is directly impacted by pre-tensile deformation at room temperature, but it is important to further investigate how the dislocation density changes when the blank is subsequently heated and quenched. XRD data is utilized to evaluate and ultimately calculate the internal dislocation density by the Williamson–Hall (WH) method [19,20]. The dislocation density is calculated using Equation (1) [21]:

(1) $$\rho =\frac{14.4e^{2}}{b^{2}}$$

where *ρ*, *e*, and *b* are the dislocation density, the micro-strain, and Burger’s vector (for ferrite, it is 0.248 nm), respectively. The micro-strain can be established using Equation (2) based on the test results of the (110)α, (220)α, and (211)α peaks.

(2) $$\delta_{hkl}cos\theta_{hkl}=\frac{\lambda }{D}+2esin\theta_{hkl}$$

where *λ* and *D* are the wavelengths of the radiation and apparent size parameter. *θ_hkl_* is the Bragg angle of the diffraction peak. *δ_hkl_* is the physical broadening of the full width at half maximum (FWHM) of the diffraction peak, which can be calculated using Equation (3) [22]:

(3) $$\delta_{hkl}={\left(\delta_{hklm}^{2}-\delta_{hkl0}^{2}\right)}^{0.5}$$

where *δ_hklm_* and *δ_hkl0_* are the FWHM of the test specimens and standard specimens. According to Equation (2), by plotting the value of *δ_hkl_cosθ_hkl_* as a fraction of 2*sinθ_hkl_* and using the straight-line fitting method, the slope of the line is the micro-strain.

**Figure 7:**
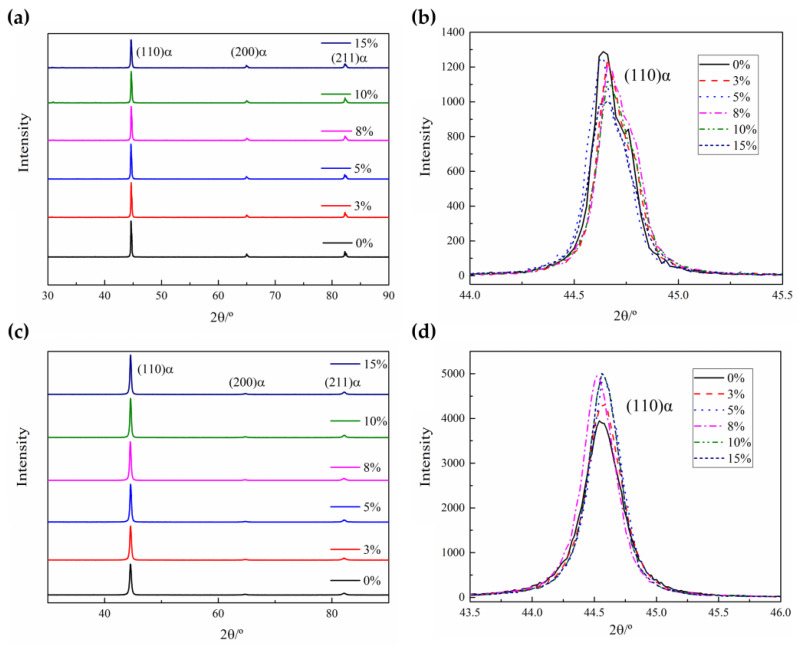
XRD patterns of the specimens with different elongations: (**a**) room-temperature specimens; (**b**) zoomed image in the range of 43.5°~46° of the room-temperature specimens; (**c**) quenched specimens; (**d**) zoomed image in the range of 43.5°~46° of the quenched specimens.

Figure 7 shows the XRD patterns of the specimens with different elongations at room temperature and after quenching. The diffraction pattern of the room-temperature specimens and quenched specimens shows three obvious α-phase diffraction peaks (Figure 7a,c). For the room-temperature specimens, the intensity of the three peaks slightly decreases and broadening occurs with the increase in elongations, especially in the (110)α main diffraction peak. This is caused by strain and lattice distortion during pre-tensile deformation at room temperature. For the quenched specimens, the intensity of the three peaks slightly increases with the increase in elongations, especially in the (110)α main diffraction peak. This is due to the change in the solid solution degree of carbon in the α-phase, and the lattice constants are different, which indicates that the martensite phase distribution is more uniform [23].

According to the WH method, the dislocation density of the specimens at room temperature increases from 0.29 × 10^16^ m^−2^ to 0.42 × 10^16^ m^−2^, and the dislocation density of the quenched specimens decreases from 1.35 × 10^16^ m^−2^ to 0.95 × 10^16^ m^−2^. Due to the shear properties of martensite, a high dislocation density is generated during phase transformation; thus, the dislocation density of the quenched specimens is higher than the room-temperature specimens. A similar reduction phenomenon in dislocation density with plastic deformation is also found in other low-carbon martensitic steels [24,25,26].

**Figure 8:**
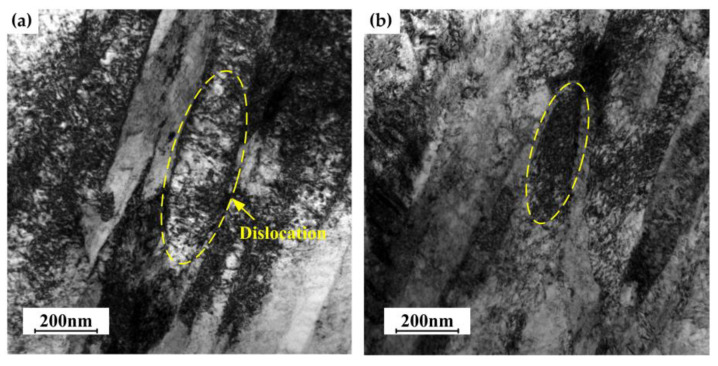
TEM image of the quenched specimens: (**a**) 0%; (**b**) 15%.

Figure 8 shows the TEM image of the 0% and 15% quenched specimens. The width of the martensite is about 200 nm, a large number of dislocations are formed, and the dislocations are intertwined with each other. No large difference is found in both images, but it appears the distribution of the dislocations slightly changes after deformation. The dislocation disperses randomly in the 0% quenched specimen but tangles each other to form a network cell structure in the 15% quenched specimen. The dislocations that formed after martensite transformation are movable dislocations and randomly distributed. However, in the deformed specimens, the dislocations shifted slightly. Some dislocations disappear during the deformation, and the remaining dislocations move to a stable position and form an entangled network cell structure, which makes the original randomly distributed dislocations become stable. This explains why the dislocation density of the quenched specimens slightly decreases with the increase in elongations.

**Figure 9:**
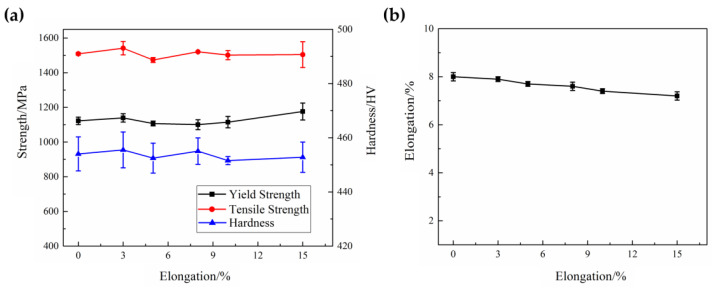
Mechanical properties of the quenched specimens with different elongations: (**a**) strength and hardness (**b**) elongation.

Figure 9 shows the mechanical properties of the quenched specimens with different elongations. The yield strength, tensile strength, and hardness results fluctuate within a specific range at each elongation. The elongation slightly decreases, because with the increase in pre-tensile deformation at room temperature, the specimens’ cross-section width and thickness slightly decrease; thus, the quenched specimens are more prone to early instability during the second tensile test. In summary, under the combined effect of the grain size and dislocation density, pre-forming does not have a great effect on the mechanical properties of the quenched blank. The overall mechanical properties all meet the hot-stamping requirements, and the results are similar to Min’s studies [18,27].

## 3. Effect of the Pre-Forming Volume on the Part’s Formability in Indirect Hot Stamping

According to the experimental results, different pre-forming does not have a great effect on the mechanical properties of the quenched blank. Thus, another important aspect of indirect hot-stamping technology is to focus on the part’s formability (forming quality) both in the pre-forming and the subsequent hot-forming stages. This paper uses a typical part as an example to discuss the effect of the different pre-forming volumes on the part’s formability. Here, the press stroke is used to define the pre-forming volume, which is the proportion of the part’s maximum forming depth.

### 3.1. Part and Finite-Element Model

**Figure 10:**
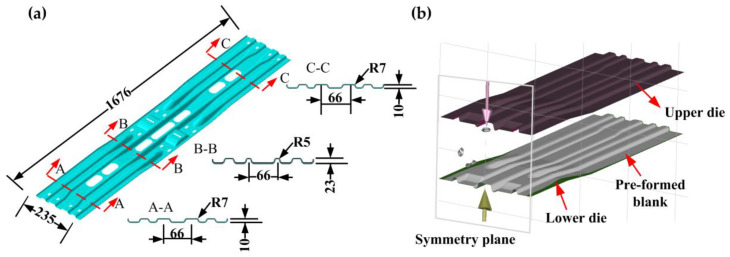
(**a**) Beam part model and (**b**) FE model.

**Figure 11:**
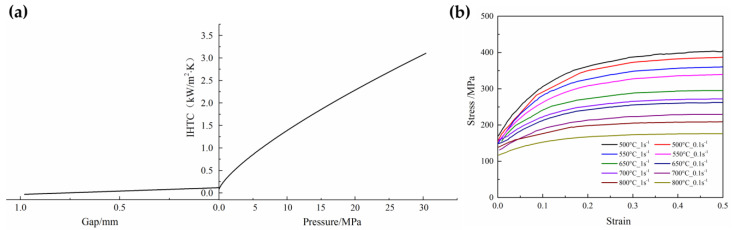
(**a**) IHTC with the pressure and gap and (**b**) stress–strain curves of 22MnB5 at different conditions.

As the main component of an equipment bracket in the bus, the beam part is shown in Figure 10a. The thickness of the beam part is 2 mm and has four concave–convex structures in the cross-section that gradually become deeper from both sides to the middle. The maximum forming depth in the middle section (Section B-B) is 23 mm, and the forming depth at both sides is 10 mm (Sections A-A and C-C). Due to the complex surface of the part, crack and wrinkle defects always occur when using direct hot stamping; thus, this paper manufactured the part by indirect hot stamping. FE software AutoForm R7 was used to carry out the numerical simulations, and the FE model is shown in Figure 10b. The upper die and lower die were set as the rigid body. The upper die was punched with the stamping speed, and the lower die was fixed in all directions. The stitching distance and meshing tolerance between the geometric surfaces were set to 0.5 mm and 0.03 mm. After meshing, there were 380,314 elements and 175,961 nodes. The detailed material information and simulation conditions are listed in Table 2. The interfacial heat transfer coefficient (IHTC) depends on the gap and the quenching force between the blank and dies, as shown in Figure 11a. The die material used was hot work steel KDAHP1. The numerical simulation included a series of hardening curves that covered the entire hot-stamping temperature, for example, as shown in Figure 11b.

To investigate the effect of the different pre-forming volumes on the part’s formability, numerical simulations were designed with different pre-forming volumes of 30%, 50%, 70%, and 90%. The maximum forming depth of the beam part is 23 mm, so the pre-forming volumes are 6.9 mm, 11.5 mm, 16.1 mm, and 20.7 mm respectively. First, the blank was formed with different pre-forming volumes at room temperature. Then, the pre-formed blank was placed into a furnace sufficiently heated to 930 °C and held for 5 min to guarantee complete austenitization. After heating, the heated pre-formed blank was removed from the furnace and transferred to the dies for further stamping and quenching. The stamping speed was 100 mm/s, the quenching force was 1000 kN, and the quenching time was 10 s. The thickness thinning result of the part is utilized as the evaluation index in actual manufacturing since it is not accurate to quantify the formability only based on the forming limit diagram (FLD) to determine whether the part is cracked or wrinkled. Therefore, this paper used the thickness thinning rate to evaluate the forming result and required the maximum thickness thinning rate of the part should be less than 20%.

### 3.2. Finite-Element Analyses and Experimental Results

**Figure 12:**
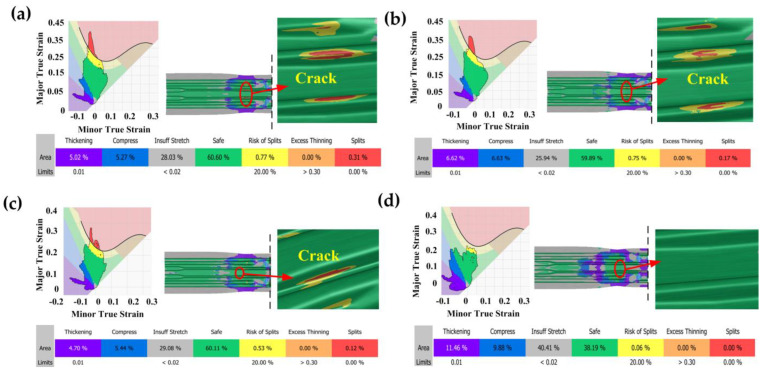
FLD results of the beam part with different pre−forming volumes in the indirect hot stamping: (**a**) 30%; (**b**) 50%; (**c**) 70%; (**d**) 90%.

Figure 12 shows the FLD results of the beam part with different pre-forming volumes in the indirect hot stamping. When the pre-forming volume is less than 90%, cracks occur in the sidewall reign of the concave–convex structure in the middle of the parts, and the maximum thickness thinning rate is over 20%. However, with the increase in the pre-forming volumes from 30% to 70%, the maximum thickness thinning rate decreases from 30.1% to 26.5% (Figure 12a–c) and the cracking area decreases. Only when the pre-forming volume is 90% (Figure 12d) is there no crack and the maximum thickness thinning rate is 19.1%, which meets the hot-stamping requirements.

**Figure 13:**
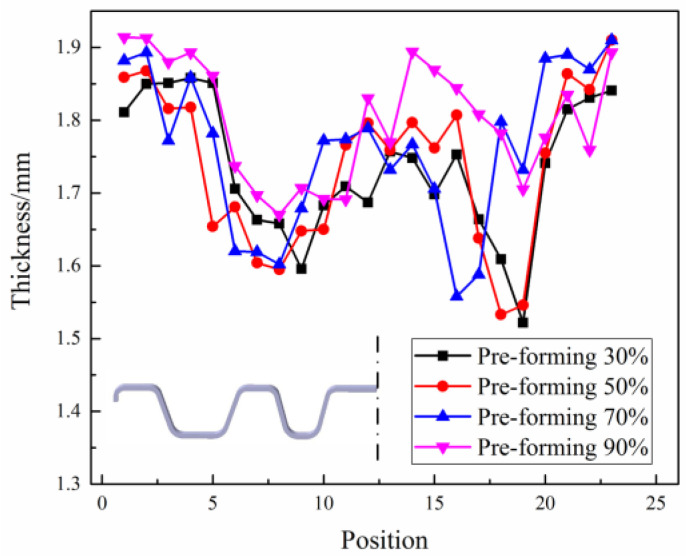
Thickness distribution results of the part with different pre-forming volumes.

The thickness distribution results in the middle section of the beam part are shown in Figure 13. When the pre-forming volume is from 30% to 70%, the overall thickness values of the parts are low and have an excessive thinning area. In comparison, when the pre-forming is 90%, the thickness distribution of the part is more uniform, and there is no excessive thinning area, which implies better formability. Indirect hot-stamping technology is more advantageous in manufacturing complex parts such as the beam part because most plastic deformation of the part is completed in the pre-forming stage. With the increase in the pre-forming volumes, the size and shape of the pre-formed blank gradually become close to the final part’s size, then the deformation during the subsequent hot-forming stage is small, the role of the subsequent hot forming is more about shaping and forming local features of the pre-formed blank. Thus, the thickness distribution is more uniform and can avoid excessive thinning caused by the large deformation at high temperatures. Therefore, with the increase in the pre-forming volumes, the part will achieve better formability.

**Figure 14:**
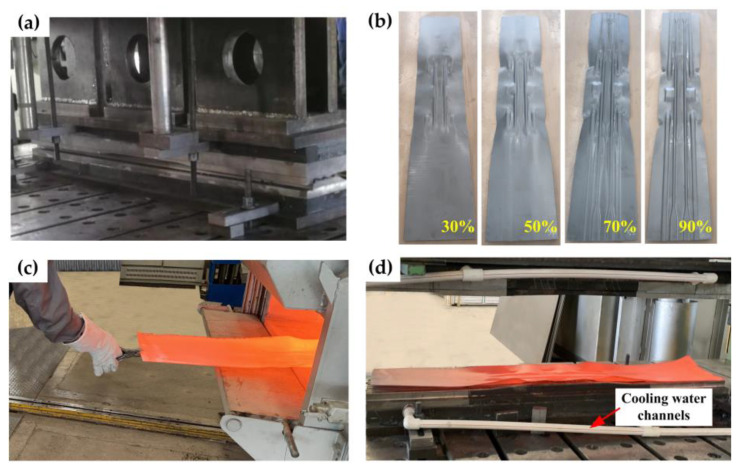
Indirect hot-stamping experiment: (**a**) pre-forming; (**b**) pre-formed blanks after different pre-forming; (**c**) heating and transferring; (**d**) forming and quenching.

Figure 14 shows the indirect hot-stamping experiment process, the process parameters were in agreement with the numerical simulations. The blank was first pre-formed with different pre-forming volumes of 30%, 50%, 70%, and 90%. Then, the pre-formed blank was transferred into the furnace for austenitization and later put in the dies for further forming and quenching. It can be seen that in Figure 14b, with the increase in the pre-forming volumes, the blank will approach the final part size, and there is no crack or wrinkle defect on the pre-formed blanks.

**Figure 15:**
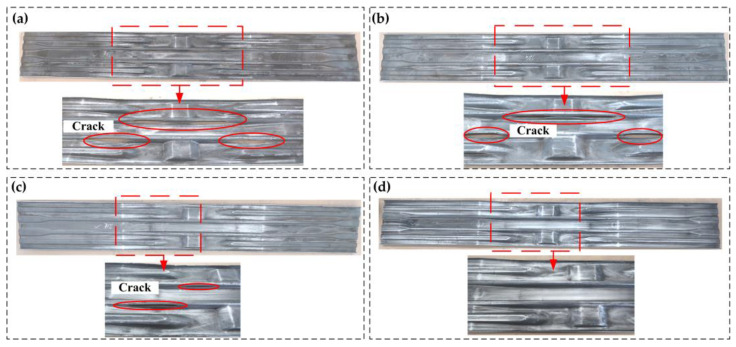
Beam test part with different pre-forming volumes in the indirect hot stamping: (**a**) 30%; (**b**) 50%; (**c**) 70%; (**d**) 90%.

Figure 15 shows the beam test part with different pre-forming volumes in the indirect hot stamping. When the pre-forming volume is between 30% and 70% (Figure 15a–c), the side wall of the convex–convex structure in the middle of the test parts experiences various degrees of crack. When the pre-forming volume is 30% (Figure 15a), the test part has the largest cracking area. When pre-forming is between 50% and 70%, the test parts also occur cracking, but the cracking area decreases. When pre-forming is 90%, the test part has good formability and there is no cracking area. Thus, it is verified that increasing the pre-forming volume can improve the part’s formability in indirect hot stamping. Additionally, the experimental results are the same as the numerical simulation results.

**Figure 16:**
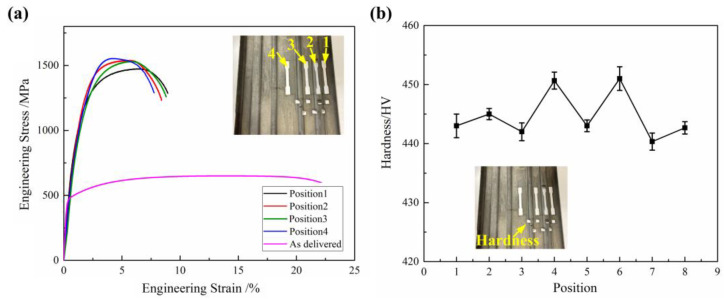
Mechanical properties of the 90% pre-forming test part: (**a**) engineering stress–strain curve; (**b**) hardness distribution.

Figure 16 shows the mechanical properties results of the 90% pre-forming test part. It can be seen that the tensile strength of the part after indirect hot stamping has significantly improved compared to the delivered blank. The tensile strength at different positions is slightly different, but the average tensile strength is above 1500 MPa, and the average elongation is about 8%. The average hardness is above 440 HV, and the sidewall region’s hardness is lower than the flat region because the sidewall region had a smaller quenching rate. Overall, the mechanical properties of the 90% pre-forming test part are meeting the hot-stamping requirements.

## 4. Conclusions

This paper reveals the evolution of the microstructure and mechanical properties of 22MnB5 steel in indirect hot stamping and provides guidance for better manufacturing parts in indirect hot stamping.

(1) In terms of the microstructure, with the increase in pre-forming, the average grain size at room temperature gradually decreases from 6.2 μm to 5.6 μm. After austenitization, the average austenite grain size slightly decreases with the increase in pre-forming from 44.5 μm to 40.5 μm, and the austenite grain distribution is more uniform. Moreover, after quenching, the martensite has the same distribution rule as the austenite grain.
(2) Although the dislocation density of the quenched specimens slightly decreases from 1.35 × 10^16^ m^−2^ to 0.95 × 10^16^ m^−2^ with the increase in pre-forming, and the mechanical properties of the quenched blank are not greatly affected because the grain size is also refined in the pre-forming range. It is demonstrated that pre-forming does not have a great effect on the mechanical properties of the quenched blank.
(3) When using the press stroke as a measurement of the pre-forming volume, the maximum thickness thinning rate of the final beam part gradually decreases, and the thickness distribution is more uniform with the increase in the pre-forming volume. When the pre-forming volume is 90%, the final beam part has better formability and more uniform thickness distribution results.

## Figures and Tables

**Table 1:** Chemical composition of the 22MnB5 steel (wt%).

C	Mn	Si	Cr	B	P	S	Ti	Al
0.23	1.12	0.26	0.13	0.003	0.012	0.002	0.035	0.036

**Table 2:** Material properties and simulation conditions.

Material	22MnB5
Young’s modulus (GPa)	212
Poisson’s ratio	0.3
Density (kg/m^3^)	7.89 × 10^−3^
**Simulation condition**	
Die temperature (°C)	25
Friction coefficient	0.3
IHTC (mW/mm^2^k)	The function of pressure/gap
Heat transfer to ambient (mW/mm^2^k)	20 °C-0.002 950 °C-0.075

## Data Availability

Not applicable.
